# Telerehabilitation proposal of mind-body technique for physical and psychological outcomes in patients with fibromyalgia

**DOI:** 10.3389/fphys.2022.917956

**Published:** 2022-08-26

**Authors:** Teresa Paolucci, Alessandro de Sire, Martina Ferrillo, Dania di Fabio, Aurora Molluso, Antonia Patruno, Mirko Pesce, Carlo Lai, Chiara Ciacchella, Aristide Saggino, Francesco Agostini, Marco Tommasi

**Affiliations:** ^1^ Department of Oral, Medical and Biotechnological Sciences, Physical Medicine and Rehabilitation, University G. D’Annunzio, Chieti, Italy; ^2^ Department of Medical and Surgical Sciences, University of Catanzaro “Magna Graecia”, Physical and Rehabilitative Medicine Unit, University Hospital “Mater Domini”, Catanzaro, Italy; ^3^ Department of Health Sciences, University of Catanzaro “Magna Graecia”, Catanzaro, Italy; ^4^ Italian Association of Fibromyalgia Syndrome (AISF), L’Aquila, Italy; ^5^ Department of Oral, Medical and Biotechnological Sciences, Course of Studies in Physiotherapy, University G. D'Annunzio, Chieti, Italy; ^6^ Department of Medicine and Aging Sciences, University G. D’Annunzio, Chieti, Italy; ^7^ Department of Dynamic and Clinical Psychology, Sapienza University of Rome, Rome, Italy; ^8^ Department of Anatomical and Histological Sciences, Legal Medicine and Orthopedics, Sapienza University, Rome, Italy

**Keywords:** fibromyalgia, telemedicine, psychology, pain, rehabilitation, physical therapy, fatigue

## Abstract

Fibromyalgia (FM) syndrome is characterized by the close correlation of chronic widespread pain and other non-pain related symptoms. Aim of this study was to investigate whether telerehabilitation that provides physical and psychological support services of the mind-body techniques can affect the clinical profile and pain relief of FM patients. The study included twenty-eight female FM patients, mean aged 56.61 ± 8.56 years. All patients underwent a rehabilitation treatment (8 sessions, 1/week, 1 h/each) through Zoom platform, with the following principles of rehabilitation treatment: Anchoring to a positive emotion; listen and perceive your “own” body; conscious breathing; improve interoceptive awareness; relax. All patients then underwent clinical assessment of the physical distress and fear of movement for the Numeric Rating Scale (NRS); the Fatigue Assessment Scale (FAS); the Fear Avoidance Belief Questionnaire (FABQ); with measures of physical and mental disability for the Fibromyalgia Impact Questionnaire (FIQ); the 12-Items Short Form Survey; the Resilience Scale for Adults and the Coping Strategies Questionnaire-Revised. The evaluations were performed at T0 (baseline), T1 (after 8 weeks of treatment), and T2 (after 1 month of follow-up). The main finding was that telerehabilitation reduced physical and mental distress, fear, and disability (*p* < 0.001). Resilience and coping ability were less affected by the rehabilitative treatment. Our attempt of mind-body technique telerehabilitation has shown good results in the improvement of painful symptoms and quality of life for the FM patients but showed fewer positive impacts for the resilience and coping abilities aspects.

## 1 Introduction

Fibromyalgia syndrome (FM) is characterized by the close correlation of chronic widespread pain and other non-pain related symptoms, such as fatigue, poor sleep, and cognitive disturbances, which are variable during the disease. Surely, the physical and psychological distress have a continuous distribution in the FM characterizing the clinical profile of each fibromyalgia patient (Kaltsas et al., 2020). It occurs in all populations throughout the world, with prevalence between 2% and 4% (Häuser and Fitzcharles, 2018).

Although the etiology of this condition is unknown, its onset and maintenance appear to be explained by a complex interplay between genetic/biological and psychosocial factors, which negatively affect the course of the disease and the quality of life (Stisi et al., 2008; Tesio et al., 2018; Varallo et al., 2021a).

It has been suggested that a multidimensional approach is the best option for the care and cure of FM patients that include pain management, pharmacological therapies, patient education, rehabilitative approach and psychological interventions are recommended. Since evidence suggests that only a small portion of FM patients continue to follow their doctors’ prescriptions for pharmacotherapy over time due to diminished effectiveness, side effects, and costs, non-pharmacological intervention, as rehabilitative exercise and mind-body technique, has recently gained attention. For example, low to moderate aerobic and muscle strengthening exercises represent an effective way of reducing pain in FM patients. (Macfarlane et al., 2017; Sosa-Reina et al., 2017; Araújo and DeSantana, 2019; Maffei, 2020).

The relevant role of pain management, also psychological factors should be taken into account, as psychological factors significantly contribute to both symptomatology and functioning in the treatment response in chronic pain patients.

Fear of movement, in fact, is associated with worse physical functioning and higher levels of pain intensity, while protective factors such as higher resilience and functional coping skills are associated with lower symptom burden and better adjustments (Rodero et al., 2011; Varallo et al., 2020; Varallo et al., 2021b). Thus, a psychological intervention that considers these aspects is necessary, in a multidisciplinary and interdisciplinary rehabilitation context. Furthermore, the proprioceptive rehabilitative exercise and the awareness of movement and posture were effective on pain and psychological distress, based on the aspects of perceptual incoherence that occurs in chronic painful pathologies such as FM (Paolucci et al., 2016; Paolucci et al., 2022).

Also mind body interventions (Theadom et al., 2015), that are based on the holistic principle that mind, body and behaviors are all interconnected, could be good therapeutic strategies to improve psychological and physical well-being in FM patients.

For example, guided imagery relaxation therapy could be incorporated as part of FM treatment with breathing technique to improvement in physiological reactivity to a standard laboratory stressor (Kozasa et al., 2012; Schmidt et al., 2012; Onieva-Zafra et al., 2015).

Patients with FM should be monitored in terms of psychological distress, especially during the pandemic period (Haugmark et al., 2019). The COVID-19 pandemic period has negatively affected the management of chronic health conditions such as fibromyalgia, with important consequences on the quality of life of affected patients. (Casale et al., 2019; Agostini et al., 2021; Cankurtaran et al., 2021; de Sire et al., 2021).

Also, during the pandemic period of the SARS-CoV-2, the provision of face-to-face rehabilitation therapies was, often, not possible for lockdown and restrictions to prevent the spread of COVID-19 and reduce the infection risk (Salaffi et al., 2021).

The, telerehabilitation architecture, with separate units for delivery of therapeutic protocols, recording of performance, and providing of feedback and monitoring progress could be an important resource (Mbada et al., 2019).

The FM patient during telerehabilitation could be evaluated along the coordinates of these three dimensions (*stress, resilience, and coping*) in order not only to verify the effectiveness of the therapy, but to understand which are the patient’s weak points on which to intervene (Meulenberg et al., 2022).

Therefore, the telerehabilitation has just represented an important rehabilitation resource in many chronic painful pathologies as spine pain, neurological diseases, facial palsy, etc. (de Sire et al., 2022a) A recent study by Hernando-Garijo et al. (2021) reported a telerehabilitation protocol of 15 weeks, with 2 sessions per week, based on aerobic exercise guided by video, that proved to be effective in reducing pain and psychological distress.

From these premises, our aim was to evaluate the effects of a telerehabilitation on pain severity, physical and psychological aspects in a sample of patients with fibromyalgia. Our hypothesis was that a telerehabilitation program based on body awareness exercises in movement and muscle stretching, linked to mindfulness “*here and now*” strategies, could reduce pain and improve the main symptoms of FM which are variable during a period of the disease of mobility and social restrictions imposed by the COVID-19 pandemic (Casale et al., 2019; de Sire et al., 2021; Salaffi et al., 2021; Meulenberg et al., 2022).

Therefore, the purpose of this study was to investigate whether telerehabilitation that provides physical and psychological support services of the mind-body techniques can affect the clinical profile and pain relief in a sample of patients with FM.

## 2 Materials and methods

### 2.1 Participants

We included patients with diagnosis of fibromyalgia, satisfying the American College of Rheumatology criteria (Wolfe Wolfe et al., 2010), recruited by patients’ association—IAFS, considering the following inclusion criteria: Age of 18–60 years; a score of >3 on the Numeric Rating Scale (NRS), in the last 3 months and baseline condition of sedentary lifestyle with no or irregular physical activity, a pharmacotherapy regimen stable for at least 3 months before the patient began treatment.

The exclusion criteria were as follows: The presence of concomitant autoimmune diseases, psychiatric disorders (as a diagnosis of major depression), or other causes of chronic pain; other diseases that prevented physical loading; surgery of the spine; vertebral fractures; sciatic pain; tumors; and enrollment in another type of physical therapy program. Patients were excluded if they had comorbidities, such as cardiovascular risk factors, previous myocardial infarction, lower extremity arterial disease, major neurological problems, diabetes, gastrointestinal disease, chronic respiratory disease, kidney disease, and severe poor vision.

All patients were given explanations about the study by the referring psychologist and by the physician speacialized in physical medicine and rehabilitation through an on-line one-to-one interview.

### 2.2 Outcome measures

Self-report questionnaires were administered to analyze the evolution of physical and psychological conditions in patients during the study. The different measures were divided into four groups: 1) Physical distress and fear of movement, 2) physical and mental disability, 3) resilience and 4) coping ability. The evaluations were performed before treatment (T0 = baseline), at the end of the rehabilitative treatment (T1 = 8 sessions 1/week, 1 h/each), and after 1 months of follow-up (T2).

#### 2.2.1 Measures of physical distress and fear of movement

*Numeric Rating Scale* (NRS) for pain. This scale consists of one specific item, which measures the level of pain suffered by the patients. Score is from 0 (no pain) to 10 (maximum pain) and the higher the score is the higher the level of pain (Hjermstad et al., 2011).

*Fatigue Assessment Scale* (FAS). This scale consists of ten items with a 5 points Likert scale. The higher the score, the higher level of fatigue or distress felt by the patient. The scale is divided into two subscales, each with 5 items: the Physical FAS (P-FAS) which measures physical fatigues and the Mental FAS (M-FAS) which measures cognitive fatigue (De Vries et al., 2004).

*Fear Avoidance Belief Questionnaire* (FABQ). A self-report questionnaire that consists of 16 items with a 6-point Likert scale. The total score ranges from a minimum of 0 to a maximum of 96. The higher the score, the higher is a patient’s fear and tendency to avoid movement (Monticone et al., 2012).

#### 2.2.2 Measures of physical and mental disability

*Fibromyalgia Impact Questionnaire* (FIQ). This scale consists of 20 items with a 5 points Likert scale. The higher the score, the higher the level of disability by the patient. This scale also includes a subscale for measuring the level of anxiety experienced along with the disability (Anxiety-FIQ or A-FIQ) (Sarzi Sarzi-Puttini et al., 2003).

*12-Item Short Form Survey* (SF-12). This scale is a self-reported outcome measure assessing the impact of health on an individual’s everyday life. The lower the score, the higher the level of disability experienced by the patients. The scale includes two types of disability scores: A physical (PSF-12) and a mental score (MSF-12) (Kodraliu et al., 2001).

#### 2.2.3 Measures of resilience

Patients’ resilience was measured with the *Resilience Scale for Adults*. This scale examines intrapersonal and interpersonal protective factors presumed to facilitate adaptation to psychosocial adversities and consists of 33 items with a 7 points Likert scale. The higher the score, the higher the patience’s resilience. The scale includes six subscales for intrapersonal and interpersonal factors of resilience. The intrapersonal factor subscales are: Perception of Self (RSA-PS) which evaluates the individual’s confidence in her/his abilities; Planned Future (RSA-PF) which assesses the individual’s ability to make plans for future; Social Competence (RSA-SC) which assesses the individual’s ability to socialize with others and Structured Style (RSA-SS) which evaluates the individual’s ability to organize her/his activities. The interpersonal factor subscales are: Family Cohesion (RSA-FC) which evaluates the support received by the individual from her/his family and Social Resources (RSA-SR) which evaluates the support received by the individual from her/his friends or others (Friborg et al., 2003; Bonfiglio et al., 2016).

#### 2.2.4 Measures of coping ability

Patients’ coping ability was measured with the *Coping Strategies Questionnaire-Revised* (CSQR) that consists of 27 items with a 6 points Likert scale (Monticone et al., 2014). The higher the score, the higher is a patient’s ability to cope with stress and disease generated by the illness. The scale includes six subscale that are: Distraction (CSQR-DS), which assesses the individual’s ability to think about things or activities that can distract her/him from the problem; Catastrophizing (CSQR-CT) which evaluates the tendency of the individual to see her/his situation worsen in the future; Ignoring Pain Sensations (CSQR-IPS) that evaluates the individual’s ability to ignore painful sensations; Distancing From Pain (CSQR-DFP) which evaluates the individual’s ability to imagine pain as a sensation outside from her/his body; Coping Self-Statements (CSQR-CSS) that evaluates the individual’s ability to continue her/his activity despite physical pain; and Praying (CSQR-Pray) that assesses the tendency of the individual to ask for help from a supernatural entity. The scales were administered through the google-Moodle platform.

#### 2.2.5 Telerehabilitation plan

The rehabilitation therapies were performed using the Zoom platform in collaboration with the Italian Association of Fibromyalgia Syndrome (AISF), Abruzzo Section, L’Aquila, Italy and through the google-moodle platform the above mentioned scales were made available. All patients made a synchronous connection by creating a virtual class of participants: the rehabilitation session was performed by the physiotherapist who showed all the phases of the exercises in sync with the patients. Together with the physiotherapist, the physiatrist and the psychologist of the IAFS (for any doubts or questions *via* chat) were present at the session, with the camera turned off or on as desired.

The 8 treatment sessions were preceded by one on-line meeting, that acquainted the patients with the rehabilitation process and the contents of the program. In addition, a chat-telephone group was created, available to patients where both the physiotherapist, the physiatrist and the psychologist made themselves available. An optional end session was provided to those who missed a session during the course.

Patients were provided with paper support, with photos and explanations of the exercises, in order to be able to repeat them at least one more time during the week independently.

The principles of rehabilitation treatment were: 1) anchoring to a positive emotion through the choice of a color; 2) “here and now”: listen and perceive your “own” body in motion; 3) conscious breathing; 4) “close your eyes”: improve interoceptive awareness during exercise; 5) relaxation: Breathe, moving slowly and without pain (Safran and Sanda, 2015; Kawai et al., 2020).

At the beginning of each session, the physiotherapist reminded the patients of the importance of having comfortable clothing, bare feet or wear non-slip socks, a comfortable and peaceful environment (those who wished could insert a background music of their choice). Patients were asked to have the camera on and the microphone off. Each patient could, in case of need, turn on the microphone to ask any question when switching exercise.

##### 2.2.5.1 First phase

1) Proposal of the color associated with the session. Request to the patient to visualize a scene, in an external environment, natural with pleasant memory. Maintain these positive emotions and feelings associated with the memory during the session. At the patient’s choice, metaphorically paint a part of the body with the reference color. We used 8 color stimuli: Red, orange, yellow, green, blue, purple, pink, and brown.

##### 2.2.5.2 Second phase

i) About 5–15 min of warm-up, with a light walk on the spot, then adding the movement of the arms, alternately and rhythmically.

ii) Patient in the supine position, knees bent, become aware with the phases of the breath. Then, work on conscious breathing, with eyes closed and alternate normal breathing with diaphragmatic breathing exercises.

iii)Exercises of active mobilization, with phases of isometric maintenance of posture at the end of the sequence. The patient began the exercises in a supine position, then on the side, followed by a quadrupedal position and, finally, in an upright position. All movements were proposed to the patient with open eyes and then with closed eyes, rhythmic speed with the slow rhythm of breathing and recognizing and perceiving the movement and position of the body in space. The physiotherapist, in the first sessions explained the exercises and illustrated them, then guided movement with her voice in every aspect. In the following sessions, the therapist guided with her voice and simultaneously performed the exercise together with the patients.

Each movement was repeated 5–10 times, with rest breaks of at least 1–2 min between the execution of one repetition and the next:

i) exercises for active joint mobilization of the lower and upper limbs (flexion, extension, rotation);
ii) self-mobilization exercises of the spine;
iii) “bridge” and “cat” exercise*;*
iv) lower and upper limb coordination exercise in quadrupedal position and standing position
v) exercise to maintain balance in single stance;
vi) exercise of maintaining balance in mono-podalic support with hip abduction*;* When the patient performed the movement with his eyes closed, he was asked to imagine it before performing it (motor imagery task). The sequence used was: eyes open - eyes closed - eyes open (5 repetitions) at a time.
vii) exercises of active global stretching, on the mat in the first 4 sessions and, also, in standing position in the last 4 sessions.

**FIGURE 1:**
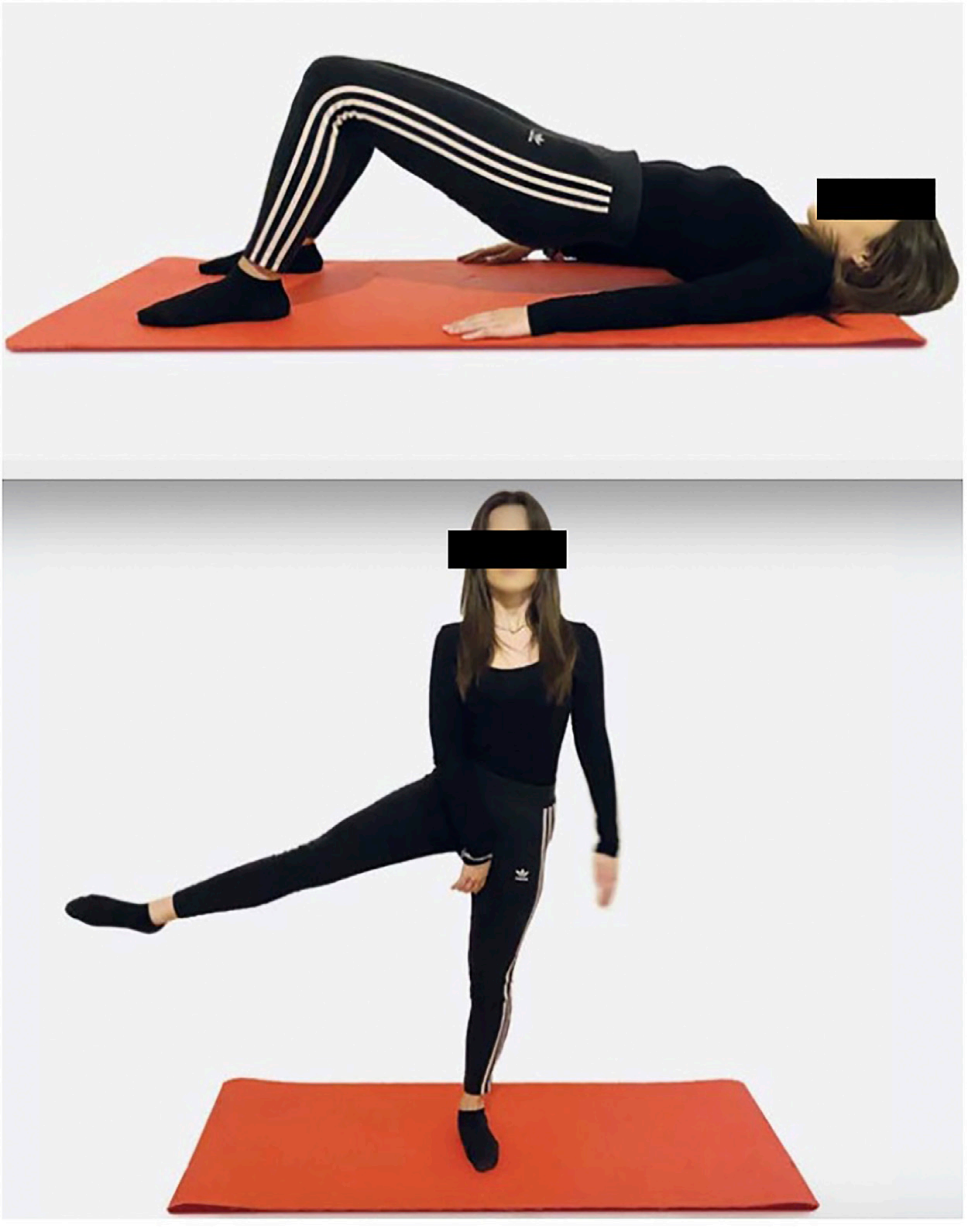
“Bridge” exercise and maintaining balance in mono-podalic support with hip abduction.

All patients were always asked to stop if they experienced pain. The physiotherapist adapted and controlled all the exercises to avoid any adverse event and to ensure patients’ safety. See Figure 1 for further details on rehabilitation exercises.

### 2.3 Statistical analysis

A multivariate analysis was performed on different measures of physical and psychological characteristics of FM patients under telerehabilitation. The aim of these analysis was double: firstly, we analyzed the temporal evolution of physical and psychological characteristics during the three periods of evaluation time (T0, T1, and T2); subsequently with multidimensional scaling applied to all physical and psychological measures, we identified the latent dimensions by which it should be possible to categorize FM patients under telerehabilitation to allow the implementation of specific intervening strategies and rehabilitative care profiling FM patients.

Subsequently, multidimensional scaling showed to be a valid method of analysis in defining rehabilitative care, profiling FM patients’ under treatment or medical intervention (Groenier et al., 2003). More in detail, grouping patients’ characteristics in latent dimensions allows an easy identification of patients because of the reduced number of dimensions and because the latent dimensions put in evidence those characteristics that have to be measured or quantified to assess patients’ disease and disability (Widiger et al., 1987).

A descriptive statistic was provided for demographic and biometric data: Mean, standard deviation, standard error, skewness, and kurtosis. Skewness and kurtosis values between −2 and 2 indicate good distribution of the data (Gravetter and Wallnau, 2014).

We analyzed the trend of the rehabilitation effects on the measures of physical distress and fear, physical and mental disability, resilience, and coping ability using the regression analysis with orthogonal polynomial coefficients was used. The polynomial coefficients for the linear trend were −1, 0, and 1, and the coefficients for the quadratic trend were −1, 2, and 1. Test was applied to determine whether the linear or quadratic trend was significant. Positive (negative) t values for the linear trend indicated a constant increasing (decreasing) effect from T0 to T2 and a maximum (minimum) effect in the middle (T1) of the time series (Trochim and Donnelly, 2006). We also estimated the effect size in relation to significant t values. The effect size is low at < 0.5, medium at < 0.8, and high at > 0.8 (Cohen, 1992).

Multidimensional scaling (MDS) was applied to define the latent dimension of patients under telerehabilitation. To define the number of latent we followed the procedure of Oganian and colleagues (Oganian et al., 2018).

Firstly, we used the hierarchical clustering between measures to define the height in correspondence of which we have the most parsimonious number of latent dimensions. Subsequently, we performed MDS on Euclidean distances of measures to analyze the grouping of the different physical and psychological variables. MDS analysis was based on the average correlation between variables estimated in T0, T1, and T2. However, we used the coefficient of determination R instead of the original correlational values *r*, because it is a more reliable values of the connection between variable (Chicco et al., 2021). The coefficient R, in the case of bivariate correlations among variables, is equivalent to the square value of the correlation and it expresses the quote of variance explained by the relation between variables. The mean of the different R values calculated in T0, T1, and T2 was estimated with the root mean square of each R values, because the root mean square is invariant to squared scores. All statistical analyses were performed in R-studio (version 1.4.1717) and R (version 4.1.1).

## 3 Results

### 3.1 Descriptive analysis

The data analyzed concerned a sample of 28 female patients with FM (mean age 56.61 years, SD ± 8.56. Initially, 36 patients were included, of which 4 withdrew due to incompatibility in time and days, 3 did not sign the informed consent, and 1 only partly participated before dropping out. Table 1 shows the descriptive data for the measures of physical distress and fear (NRS; P-FAS; M-FAS; FABQ; physical and mental disability (FIQ; A-FIQ; PSF-12; MSF-2), resilience (RSA-PS,; RSA-PF; RSA-SC; RSA-SS; RSA-FC; RSA-SR) and coping abilities (CSQR-DS; CSQR-CT; CSQR-IPS; CSQR-DFP; CSQR-SS; CSQR-Pray). Skewness and kurtosis indicate that data distributions of the variables are normal.

**TABLE 1:** Mean, standard deviation (SD), standard error (SE), skewness and kurtosis of the raw data collected from the patients under telerehabilitation (*n* = 28).

Variables grouping	Scales	Mean	SD	SE	Skewness	Kurtosis
Physical distress and fear	NRS	3.226	2.113	0.231	0.197	−0.974
	P-FAS	17.119	4.457	0.486	−0.757	0.033
	M-FAS	14.202	4.599	0.502	0.297	−0.809
	FABQ	34.393	15.718	1.715	0.117	−0.700
Physical and mental disability	FIQ	38.179	10.374	1.132	−0.206	−0.603
	A-FIQ	5.583	2.365	0.258	0.313	−0.757
	PSF-12	35.528	9.844	1.074	0.304	−0.555
	MSF-12	42.996	9.561	1.043	−0.839	0.330
Resilience	RSA-PS	24.667	3.320	0.362	−0.363	−0.468
	RSA-PF	13.333	3.582	0.391	0.157	−0.794
	RSA-SC	23.417	2.967	0.324	−0.140	0.017
	RSA-SS	17.750	1.975	0.215	0.298	−0.574
	RSA-FC	22.940	2.663	0.291	0.857	1.507
	RSA-SR	30.476	3.622	0.395	−0.426	0.223
Coping ability	CSQR-DS	19.759	6.330	0.695	−1.045	0.783
	CSQR-CT	14.786	8.367	0.913	0.028	−0.918
	CSQR-IPS	19.048	6.686	0.729	−0.483	−0.531
	CSQR-DFP	10.679	8.341	0.910	−0.053	−1.541
	CSQR-CSS	18.631	4.690	0.512	−0.921	0.361
	CSQR-Pray	9.024	4.822	0.526	−0.189	−0.920

NRS, numeric rating scale; P-FAS, physical fatigue assessment scale; M-FAS, mental fatigue assessment scale; FABQ, fear avoidance belief questionnaire; FIQ, fibromyalgia impact questionnaire; A-FIQ, anxiety fibromyalgia impact questionnaire; PSF-12, physical 12-Item Short Form Survey; MSF-12, mental 12-item short form survey; RSA, resilience scale for adults (subscales: PS, perception of self; PF, planned future; SC, social competence; SS, structured style; FC, family cohesion; SR, Social Resources); CSQR, coping strategies questionnaire-revised (subscale: DS, distraction; CT, catastrophizing; IPS, ignoring pain sensations; DFP, distancing from pain; CSS, coping self-statements; Pray = praying).

Results showed that telerehabilitation strongly reduce physical and mental distress, fear, and disability in patients, with nearly all trends (linear and quadratic) strongly significant, indicating evidence of telerehabilitation efficacy.

### 3.2 Time series analysis

Table 2 reports the statistics of the time series analysis. Results showed that telerehabilitation strongly reduce physical and mental distress, fear, and disability in patients. Nearly all trends (linear and quadratic) are strongly significant, and the high significance is evidence of telerehabilitation efficacy. Resilience and coping ability are less affected by telerehabilitation because few measures show a significant linear or quadratic trend.

**TABLE 2:** Regression analysis of linear and quadratic trends for time series (T0, T1, and T2) of scales of physical distress and fear, physical and mental disability, resilience, and coping ability. Significant values of t probabilities are reported in bold type. Negative coefficients indicate a reduction in scale scores, while positive coefficients indicate an increase in time phases.

Variables grouping	Scales	Trend	Estimated coefficient	Std. error	t value	Pr (>|t|)	Cohen’s d	Effect size level
Physical distress and fear	NRS	linear	−2.04	0.260	−7.84	**<0.001**	3.018	Large
		quadratic	−3.89	0.376	−10.40	**<0.001**	4.003	Large
	P-FAS	linear	−1.18	0.571	−2.07	**0.049**	0.797	Medium
		quadratic	−2.61	0.939	−2.78	**0.010**	1.07	Large
	M-FAS	linear	−3.04	0.760	−3.99	**<0.001**	1.536	Large
		quadratic	−4.04	0.925	−4.36	**<0.001**	1.678	Large
	FABQ	linear	−10.64	2.480	−4.28	**<0.001**	1.647	Large
		quadratic	−7.29	3.450	−2.11	**0.044**	0.812	Large
Physical and mental disability	FIQ	linear	−7.82	1.670	−4.70	**<0.001**	1.809	Large
		quadratic	−8.46	1.840	−4.59	**<0.001**	1.767	Large
	A-FIQ	linear	−0.82	0.353	−2.33	**0.028**	0.897	Large
		quadratic	−1.32	0.472	−2.80	**0.009**	1.078	Large
	PSF-12	linear	6.58	1.360	4.85	**<0.001**	1.867	Large
		quadratic	7.56	1.520	4.99	**<0.001**	1.921	Large
	MSF-12	linear	2.94	2.130	1.38	0.180		
		quadratic	5.40	1.870	2.89	**0.008**	1.112	Large
Resilience	RSA-PS	linear	−0.79	0.776	−1.01	0.320		
		quadratic	1.43	0.869	1.64	0.110		
	RSA-PF	linear	0.79	0.518	1.52	0.140		
		quadratic	2.21	0.772	2.87	**0.008**	1.105	Large
	RSA-SC	linear	0.61	0.396	1.53	0.140		
		quadratic	1.96	0.735	2.67	**0.013**	1.028	Large
	RSA-SS	linear	0.11	0.478	0.22	0.820		
		quadratic	1.39	0.475	2.93	**0.007**	1.128	Large
	RSA-FC	linear	0.25	0.468	0.53	0.600		
		quadratic	−0.46	0.710	−0.65	0.520		
	RSA-SR	linear	1.64	0.739	2.22	**0.035**	0.854	Large
		quadratic	2.21	1.410	1.57	0.130		
Coping ability	CSQR-DS	linear	−0.07	1.032	−0.07	0.940		
		quadratic	−0.07	1.122	−0.07	0.950		
	CSQR-CT	linear	−1.50	0.986	−1.52	0.140		
		quadratic	−3.43	1.890	−1.81	0.081		
	CSQR-IPS	linear	−0.86	1.206	−0.71	0.480		
		quadratic	2.00	1.450	1.38	0.180		
	CSQR-DFP	linear	2.29	1.100	2.07	**0.048**	0.797	Medium
		quadratic	1.07	1.290	0.83	0.410		
	CSQR-CSS	linear	−0.04	0.798	−0.04	0.960		
		quadratic	0.68	0.960	0.71	0.490		
	CSQR-Pray	linear	−1.86	0.541	−3.43	**0.002**	1.320	Large
		quadratic	0.14	1.006	0.14	0.890		

Note: NRS, numeric rating scale; P-FAS, physical fatigue assessment scale; M-FAS, mental fatigue assessment scale; FABQ, fear avoidance belief questionnaire; FIQ, fibromyalgia impact questionnaire; A-FIQ, anxiety fibromyalgia impact questionnaire; PSF-12, physical 12-item short form survey; MSF-12, mental 12-item short form survey; RSA, resilience scale for adults (subscales: PS, perception of self; PF, planned future; SC, social competence; SS, structured style; FC, family cohesion; SR, Social Resources); CSQR, coping strategies questionnaire-revised (subscale: DS, distraction; CT, catastrophizing; IPS, ignoring pain sensations; DFP, distancing from pain; CSS, coping self-statements; Pray = Praying).

### 3.3 Multidimensional scaling for latent dimensions in patients under telerehabilitation

**FIGURE 2:**
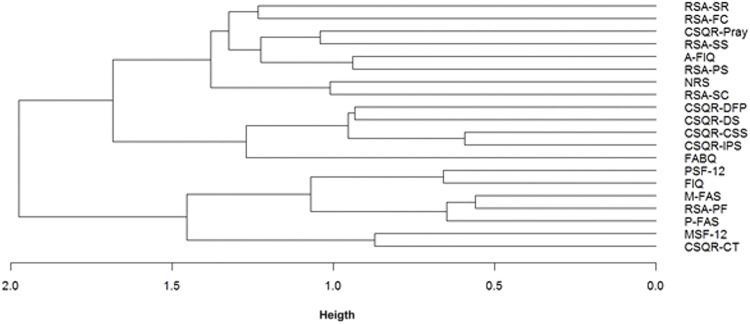
Dendrogram clustering of scales of physical distress and fear, physical and mental disability, resilience and coping ability. NRS = numeric rating scale; P-FAS = physical fatigue assessment scale; M-FAS = mental fatigue assessment scale; FABQ = fear avoidance belief questionnaire; FIQ = fibromyalgia impact questionnaire; A-FIQ = anxiety fibromyalgia impact questionnaire; PSF-12 = physical 12-Item short form survey; MSF-12 = mental 12-item short form survey; RSA = resilience scale for adults (subscales: PS = perception of self; PF = planned future; SC = social competence; SS = structured style; FC = family cohesion; SR = social resources); CSQR = coping strategies questionnaire-revised (subscale: DS = distraction; CT = catastrophizing; IPS = ignoring pain sensations; DFP = distancing from pain; CSS = coping self-statements; Pray = praying).

The root mean square of coefficients of determination and the mean of correlations among all variables for T0, T1, and T2 were reported in the Supplementary Table S1. Were used to calculate the Euclidean distance among variables. The Euclidean distances were used for the hierarchical clustering of the variables. Figure 2 is the hierarchical clustering diagram of measures, and shows at height = 1.5 that the variables are grouped into three main latent factors.

**FIGURE 3:**
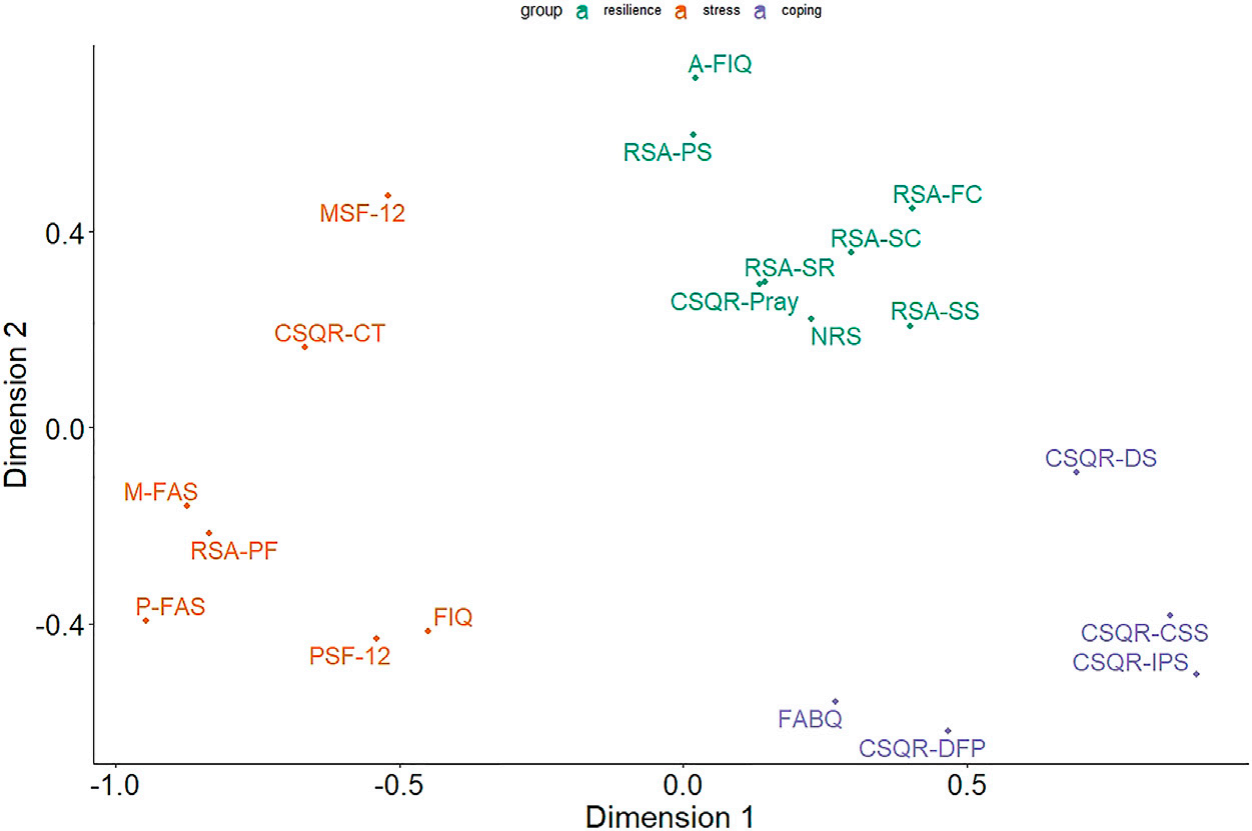
Multidimensional scaling diagram of scales of physical distress and fear, physical and mental disability, resilience and coping ability. NRS = numeric rating scale; P-FAS = physical fatigue assessment scale; M-FAS = mental fatigue assessment scale; FABQ = fear avoidance belief questionnaire; FIQ = fibromyalgia impact questionnaire; A-FIQ = anxiety fibromyalgia impact questionnaire; PSF-12 = physical 12-item short form survey; MSF-12 = mental 12-item short form survey; RSA = resilience scale for adults (subscales: PS = perception of self; PF = planned future; SC = social competence; SS = structured style; FC = family cohesion; SR = social resources); CSQR = coping strategies questionnaire-revised (subscale: DS = distraction; CT = catastrophizing; IPS = ignoring pain sensations; DFP = distancing from pain; CSS = coping self-statements; Pray = praying).

Figure 3 shows the two-dimensional structure of the latent factors, that enables to identify on the basis of the characteristics measured by the scales, a latent factor for resilience, which includes the scales NRS; A-FIQ; CSQR-Pray; RSA-PS; RSA-SR; RSA-SS; RSA-FC and RSA-SC, a latent factor for stress which includes the scales P-FAS; M-FAS; CSQR-CT; FIQ, RSA-PF; PSF-12 and MSF-12 and a latent factor for coping, which includes the scales FABQ; CSQR-DFP; CSQR-DS; CSQR-IPS, and CSQR-CSS.

## 4 Discussion

Our hypothesis was that a telerehabilitation program based on body awareness exercises in movement and muscle stretching, linked to mindfulness “Here and Now” strategies, could improve the main FM symptoms during the period of mobility restrictions imposed by the COVID-19 pandemic. Moreover, the various forms of social support by technology and a positive life approach appear to be protective with respect to the emotional and psychological distress caused by the pandemic (Salaffi et al., 2021; de Sire et al., 2022a). Considering the limitations of retrospective observational studies; however, our results are encouraging. In fact, two important issues were showed: firstly, telerehabilitation improve FM patients’ conditions, because there is a significant reduction of the level of pain, fear of movement and disability for the evaluation scales. The reduction of patients’ physical and mental distress during telerehabilitation highlights the improvement in the quality of life in patients. And even in the light of the short-term follow-up (1 month, after the end of the treatment) the patients maintained the good results achieved at T1. Also, de Sire et al. (2022a) confirmed the efficacy of telerehabilitation in FM patients, reporting improvement values on pain intensity greater than the change achieved to the MCID (Minimal Clinically Important Difference) described for patients with chronic pain (Sun et al., 2021). However, our rehabilitation protocol was mainly based on the awareness of movement, using “color” as a positive emotional anchor, having not had the possibility of monitoring the cardiorespiratory parameters, typical of aerobic training protocols albeit at low intensity and having chosen, an approach oriented to mind-body techniques, proposed with benefit in fibromyalgia patients and spine chronic pain (Tubach et al., 2005; Adler-Neal and Zeidan, 2017; Paolucci et al., 2017). Mind-body technique as mindfulness interventions can help patients cope with their stress-related experiences may reduce symptoms and improve wellbeing (Kozasa et al., 2012; Schmidt et al., 2012; Onieva-Zafra et al., 2015; Theadom et al., 2015; Meulenberg et al., 2022): these interventions are oriented to train the participants to intentionally observe thoughts, emotions, and bodily sensations as they are perceived on a moment-to-moment basis with an open, non-judgmental attitude (Day et al., 2014; Flynn, 2020).

However, resilience and coping ability, measured by RSA and CSQR subscales, are less or none affected by telerehabilitation on our sample. This could indicate that resilience and coping ability are individual characteristics probably more connected to the personality and psychological attitudes of patients, more than to the rehabilitative process. Or, from another point of view, it could be a limitation of the rehabilitation proposals not face to face with the physiotherapist and physiatrist (Tyagi et al., 2018; Vallejo et al., 2020).

Therefore, rehabilitation has a strong positive impact on patients’ quality of life, but their psychological reaction to their physical conditions is partially affected by a remote rehabilitative therapeutic process.

Considering the limitations of the retrospective observational studies, however, our results are encouraging, as in fact two important issues were shown: firstly, telerehabilitation improve FM patients’ conditions, because there was significant reduction of the levels of pain, fear of movement and disability. Secondly, the reduction of patients’ physical and mental distress (stress, resilience and coping dimensions) during the study highlights the improvement in the quality of life in patients due to the applied telerehabilitation.

The latent factor of stress includes all the negative physical and psychological aspects of rehabilitation that are, above all, the disability, sufferance and fatigue that afflict the patients. The latent factor of coping includes all the measures connected to the patient’s ability to cope or manage the stressful events during the rehabilitation, the ability to tolerate pain, continue activity and cope with the fear of therapy. In particular, FM patients showed a notable lack of self-forgiveness as problem (distress and catastrophizing) with a negative emotional status, also alexithymia related, that reduces and limits coping strategies (Moreno-Fernández et al., 2017; Di Tella et al., 2018; Bacon et al., 2021).

However, our rehabilitation protocol was mainly based on the awareness of movement, using “color” as a positive emotional anchor to induce chosen low intensity movement. An approach oriented to mind-body techniques, proposed with benefit in fibromyalgia patients and spine chronic pain (Day et al., 2014; Flynn, 2020). This in contrast to aerobic training protocols that anchor to the typical monitoring of cardiorespiratory parameters. Furthermore, the results of our research suggest that the rehabilitation process can be described as a three-dimensional space (see Figure 3) in which the first is the level of discomfort or stress related to the pathological condition, the second is the ability to resist the pain and suffering generated by the pathology and the third it is the ability to deal with the pathology.

For example, the therapy is not very effective because the patient has a low tolerance to pain and suffering, is not very resilient, or because she does not have the coping resources necessary to deal with the discomforts and difficulties: the evaluation of these important aspects by the rehabilitation team (physiotherapist, physician specializing in physical and rehabilitation medicine, psychologist and rheumatologist) would give the possibility to really adapt the rehabilitation path according to the needs of the individual patient.

It should be noted that FM share several clinical manifestations (e.g., chronic headache, stress, morning stiffness, fatigue, and mood disorders) with other diseases as temporomandibular disorders (TMD) (Chaves et al., 2016). A potential linkage between FM and TMD might be explained through the phenomenon of central sensitization and cutaneous allodynia in the distribution of the trigeminal nerve (Bevilaqua-Grossi et al., 2010). In this context, the presence of multiple comorbidities could increase the risk of developing FM and TMD and contribute to the persistence of pain. Thus, an adequate management of these comorbid patients could be a challenge for rehabilitation professionals, making necessary the role of rehabilitative approaches (e.g., physical therapy, oxygen-ozone therapy) to reduce painful symptoms and bringing comfort to the patient (Bevilaqua-Grossi et al., 2010; de Sire et al., 2022b; Ferrillo et al., 2022).

During the COVID-19 pandemic emergency, telemedicine, which includes telerehabilitation, enabled us to closely monitor FM patients over time, provide feedback, and our presented telerehabilitation tool can even offer future opportunities for cognitive-motor assessment. This important therapeutic resource should be maintained and used in parallel to traditional therapeutic pathways to more optimally serve and challenge in chronic diseases both physically and cognitively over time in future lockdowns, to provide long-term remote training and feedback (Salaffi et al., 2021; Meulenberg et al., 2022).

We are aware that this study is not free from limitations: first, the lack of the control group (for example in the waiting list), which given the contextual need for care to be provided and the lockdown period it was not possible to enroll; second, the short follow-up of re-evaluation, linked to the fact that some patients would soon have started other treatments (for example yoga courses online); third, the study design that does not define a cause and effect relationship.

Also, we did not use measurement tools (scales or questionnaires) or expert evaluations that could indicate the usefulness to implement the tool for a clinical setting. For future studies, it would be interesting to measure the feedback of the patients, but also of the caregivers on the applied telerehabilitation therapy, also using the chat unit that was manned by physiotherapist/physiatrist/psychologist during the sessions.

The literature points out that telerehabilitation has shown to provide comparable results to outpatient physical therapy and to face-to-face home rehabilitation, while reducing costs (Bini and Mahajan, 2017; Tenforde et al., 2017; Correia et al., 2018; Correia et al., 2022). Furthermore, recent studies show that these results are applicable in adults with chronic and acute musculoskeletal pain, through use fully remote digital care program with a very high retention rate and adherence level (Costa et al., 2022).

In addition, the organized architecture at the base of telerehabilitation would make it possible to deliver rehabilitation care asynchronously, and to reach the patient at any time of the day (Kushner and Sorensen, 2013; Tatta et al., 2022).

Also, it would be interesting to investigate if and how the body and mind telerehabilitation approach could be of benefit to other chronic non-communicable diseases above all in elderly patients or in psychologic confidence-lacking patients. As reported by Costa et al. (2022), the efficacy of a remote digital care program could not only reduce pain, but also improve mental health and fear-avoidant behaviors.

In conclusion, the FM remains a complex pathology with an interconnection between psychological and physical factors that might lead patients to suffer a chronic and disabling pain syndrome. Indeed, it should be taken into consideration that the mind and body could play a key role in the pathogenetic mechanism of the clinical manifestations of FM, including: Generalized chronic widespread pain, fatigue, sleep disturbances, psychological distress and cognitive alterations, joint rigidity with muscle stiffness, and tenderness.

By the present study, our attempt of mind-body technique telerehabilitation has shown good results in the improvement of painful symptoms and quality of life in the FM patient, not the same for the psychological aspects. Future studies are desirable to confirm these data in a larger sample according to the RCT model. Further developments, to re-propose more in depth, the rehabilitation protocol could provide for the support of artificial intelligence interfaces to analyze the patient’s mood and participation in the therapy with facial expressions.

## Data Availability

The raw data supporting the conclusion of this article will be made available by the authors, without undue reservation.
