# Supplementary material for: Telerehabilitation proposal of mind-body technique for physical and psychological outcomes in patients with fibromyalgia
Source: Front Physiol. 2022 Aug 26;13:917956. doi: 10.3389/fphys.2022.917956 (PMC9459112; doi:10.3389/fphys.2022.917956)
Supplement: Supplementary file 1 [file Table1.DOCX]

|  | **Supplementary Table**. Average correlations 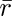 (upper triangle) and root mean square of coefficients of determination 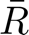 for T0, T1 and T2 of scales of physical distress and fear, physical and mental disability, resilience, and coping ability. | | | | | | | | | | | | | | | | | | | | |
| --- | --- | --- | --- | --- | --- | --- | --- | --- | --- | --- | --- | --- | --- | --- | --- | --- | --- | --- | --- | --- | --- |
|  | Outcomes | 1 | 2 | 3 | 4 | 5 | 6 | 7 | 8 | 9 | 10 | 11 | 12 | 13 | 14 | 15 | 16 | 17 | 18 | 19 | 20 |
| 1 | NRS |  | 0.19 | 0.12 | 0.01 | 0.49 | 0.16 | -0.24 | -0.12 | 0.02 | -0.11 | -0.34 | -0.10 | -0.14 | -0.16 | -0.33 | 0.12 | -0.18 | -0.15 | -0.14 | -0.06 |
| 2 | P-FAS | 0.20 |  | 0.73 | 0.30 | 0.65 | 0.14 | -0.71 | -0.41 | 0.11 | -0.67 | 0.10 | 0.07 | 0.01 | -0.25 | 0.12 | 0.46 | -0.02 | -0.27 | 0.16 | -0.08 |
| 3 | M-FAS | 0.15 | 0.74 |  | 0.17 | 0.45 | 0.13 | -0.44 | -0.53 | 0.25 | -0.72 | 0.21 | 0.11 | 0.08 | -0.28 | 0.19 | 0.54 | 0.05 | -0.21 | 0.10 | 0.35 |
| 4 | FABQ | 0.12 | 0.32 | 0.19 |  | 0.27 | -0.13 | -0.49 | -0.01 | -0.10 | -0.35 | -0.13 | -0.17 | -0.13 | -0.14 | -0.15 | 0.01 | -0.41 | -0.44 | -0.44 | -0.19 |
| 5 | FIQ | 0.49 | 0.65 | 0.45 | 0.30 |  | 0.14 | -0.65 | -0.21 | -0.03 | -0.44 | -0.19 | -0.21 | 0.03 | -0.09 | -0.16 | 0.33 | -0.30 | -0.31 | -0.07 | -0.27 |
| 6 | A-FIQ | 0.20 | 0.18 | 0.13 | 0.13 | 0.15 |  | 0.09 | -0.39 | 0.40 | -0.23 | -0.09 | 0.32 | 0.19 | -0.27 | -0.23 | 0.43 | 0.06 | 0.07 | -0.11 | 0.19 |
| 7 | PSF-12 | 0.27 | 0.71 | 0.44 | 0.51 | 0.65 | 0.16 |  | 0.08 | 0.11 | 0.48 | 0.24 | 0.13 | 0.16 | 0.13 | 0.03 | -0.28 | 0.19 | 0.25 | -0.04 | 0.26 |
| 8 | MSF-12 | 0.22 | 0.41 | 0.53 | 0.09 | 0.30 | 0.39 | 0.12 |  | -0.36 | 0.52 | 0.08 | -0.24 | -0.04 | 0.24 | 0.10 | -0.41 | -0.14 | 0.04 | 0.02 | -0.25 |
| 9 | RSA-PS | 0.28 | 0.17 | 0.28 | 0.12 | 0.18 | 0.40 | 0.24 | 0.39 |  | -0.27 | 0.05 | 0.41 | -0.15 | 0.02 | 0.06 | 0.24 | 0.21 | 0.07 | 0.13 | 0.31 |
| 10 | RSA-PF | 0.11 | 0.67 | 0.72 | 0.35 | 0.44 | 0.25 | 0.48 | 0.53 | 0.32 |  | -0.04 | -0.05 | -0.13 | 0.25 | 0.15 | -0.48 | 0.13 | 0.34 | 0.10 | -0.11 |
| 11 | RSA-SC | 0.34 | 0.18 | 0.24 | 0.13 | 0.22 | 0.18 | 0.24 | 0.15 | 0.16 | 0.05 |  | 0.01 | 0.24 | 0.06 | 0.32 | -0.02 | 0.02 | -0.16 | 0.08 | 0.11 |
| 12 | RSA-SS | 0.23 | 0.16 | 0.11 | 0.17 | 0.36 | 0.33 | 0.28 | 0.31 | 0.43 | 0.19 | 0.20 |  | -0.08 | -0.20 | 0.12 | -0.01 | 0.45 | 0.32 | 0.31 | 0.33 |
| 13 | RSA-FC | 0.17 | 0.12 | 0.11 | 0.16 | 0.10 | 0.20 | 0.18 | 0.07 | 0.25 | 0.14 | 0.27 | 0.21 |  | -0.14 | -0.17 | 0.05 | -0.15 | -0.22 | -0.09 | 0.13 |
| 14 | RSA-SR | 0.16 | 0.26 | 0.32 | 0.18 | 0.15 | 0.32 | 0.16 | 0.28 | 0.11 | 0.26 | 0.10 | 0.24 | 0.20 |  | 0.24 | -0.02 | 0.10 | 0.06 | 0.22 | -0.01 |
| 15 | CSQR-DS | 0.33 | 0.16 | 0.23 | 0.21 | 0.21 | 0.25 | 0.15 | 0.15 | 0.18 | 0.17 | 0.34 | 0.27 | 0.19 | 0.34 |  | 0.01 | 0.49 | 0.50 | 0.42 | 0.27 |
| 16 | CSQR-CT | 0.17 | 0.50 | 0.57 | 0.05 | 0.38 | 0.44 | 0.35 | 0.47 | 0.25 | 0.48 | 0.08 | 0.15 | 0.11 | 0.15 | 0.03 |  | -0.17 | -0.21 | -0.05 | 0.32 |
| 17 | CSQR-IPS | 0.25 | 0.02 | 0.15 | 0.41 | 0.32 | 0.07 | 0.20 | 0.16 | 0.21 | 0.13 | 0.11 | 0.46 | 0.19 | 0.21 | 0.51 | 0.18 |  | 0.72 | 0.77 | 0.31 |
| 18 | CSQR-DFP | 0.17 | 0.29 | 0.32 | 0.44 | 0.32 | 0.12 | 0.27 | 0.09 | 0.11 | 0.35 | 0.16 | 0.34 | 0.23 | 0.11 | 0.51 | 0.29 | 0.72 |  | 0.43 | 0.25 |
| 19 | CSQR-CSS | 0.17 | 0.17 | 0.10 | 0.44 | 0.21 | 0.18 | 0.07 | 0.11 | 0.16 | 0.11 | 0.13 | 0.31 | 0.10 | 0.26 | 0.45 | 0.12 | 0.77 | 0.43 |  | 0.10 |
| 20 | CSQR-Pray | 0.09 | 0.08 | 0.36 | 0.23 | 0.27 | 0.20 | 0.27 | 0.33 | 0.33 | 0.14 | 0.12 | 0.35 | 0.26 | 0.25 | 0.27 | 0.33 | 0.31 | 0.26 | 0.11 |  |
| Note: NRS = *Numeric Rating Scale;* P-FAS = Physical *Fatigue Assessment Scale;* M-FAS = Mental *Fatigue Assessment Scale; FABQ = Fear Avoidance Belief Questionnaire; FIQ = Fibromyalgia Impact Questionnaire; A-FIQ = Anxiety Fibromyalgia Impact Questionnaire; PSF-12 = physical 12-Item Short Form Survey; MSF-12 = mental 12-Item Short Form Survey;* RSA = *Resilience Scale for Adults* (subscales: PS = Perception of Self; PF = Planned Future; SC = Social Competence; SS = Structured Style; FC = Family Cohesion; SR = Social Resources); CSQR = *Coping Strategies Questionnaire-Revised* (subscale: DS = Distraction; CT = Catastrophizing; IPS = Ignoring Pain Sensations; DFP = Distancing From Pain; CSS = Coping Self-Statements; Pray = Praying). | | | | | | | | | | | | | | | | | | | | | |
